# Three-dimensional label-free imaging throughout adipocyte differentiation by stimulated Raman microscopy

**DOI:** 10.1371/journal.pone.0216811

**Published:** 2019-05-21

**Authors:** Maria Antonietta Ferrara, Angela Filograna, Rajeev Ranjan, Daniela Corda, Carmen Valente, Luigi Sirleto

**Affiliations:** 1 National Research Council (CNR), Institute for Microelectronics and Microsystems, Naples, Italy; 2 National Research Council (CNR), Institute of Protein Biochemistry, Naples, Italy; Pennsylvania State Hershey College of Medicine, UNITED STATES

## Abstract

Lipid droplets are lipid-storage organelles with a key role in lipid accumulation pathologies such as diabetes, obesity and atherosclerosis. Despite their important functions many aspects of lipid droplets biology are still unknown. This is partially due to the current use of exogenous labels to monitor their formation and remodelling by invasive imaging methods. Here, we apply stimulated Raman scattering microscopy to acquire images with high spatial resolution along with resolving capabilities of lipids and proteins and three-dimensional sectioning. Our images and data analysis demonstrate an increase in the number of large (>15μm^2^) lipid droplets in human adipocyte cells during differentiation process. In addition, spatially-resolved maps of lipids and proteins inside cells and three dimensional reconstructions of lipids at the initial and final steps of adipocyte differentiation are reported, too.

## Introduction

Lipids play key role in cellular physiology as structural components of biological membranes, biosynthetic precursors, signal transducers and energy storage [1]. Mammalian cells store excess of lipids molecules into specialized intracellular organelles, called lipid droplets (LDs). LDs, also known as adiposomes, are ubiquitously conserved from yeast to mammals and are involved in maintaining lipid homeostasis through lipid synthesis, metabolism, and transportation. Based on the control on these important cellular functions, LDs are closely associated with human disease such as dyslipidemia, lipodystrophy (Familial partial lipodystrophy: FGL), diabetes, obesity, fatty liver diseases (e.g. liver steatosis and cirrhosis, Non-alcoholic fatty liver disease), atherosclerosis, heart diseases (hypertrophic cardiomyopathy, or even heart failure) and cancer (hepatocellular carcinoma) [2,3]. This is the reason why in the last decade great attention has been reserved to LD biology. LDs consist of a triacylglycerol and sterol ester neutral lipid core surrounded by a single phospholipid monolayer decorated by embedded proteins that regulate lipid accumulation and mobilization. Proteomic analyses of isolated LDs identified more than one hundred of LD-associated proteins including lipid metabolism enzymes, membrane trafficking and cell signaling proteins, as well as, structural proteins [4]. Of note, these identified proteins bind to LDs across species from yeast to mammals indicating that LD is a highly conserved organelle [4]. The accepted mechanisms by which LDs grow is based on the nucleation of LDs from the Endoplasmic Reticulum (ER); there diacylglycerol starts to accumulate and recruits perilipin family members as structural LD proteins to the ER sites where then LDs bud off [5]. Seipin and the fat storage-inducing transmembrane proteins FIT1 and FIT2 cooperate for the early assembly of LDs at the ER. After ER release, LDs undergo a maturation process that is controlled by changes in the composition of the coated proteins. Perilipin proteins coordinate the access of lipases to the lipid substrates dynamically mobilizing the lipid contents following the different cellular conditions/ requirements [6].

The sizes of LDs vary from tens of nm to tens of μm in diameter [7,8]. In adipocytes, the so called “professional” fat storing cells, LDs can reach a diameter of 100 μm or even more. In these cells, LDs grow, in part, by fusion of smaller droplets and although this fusion mechanism is poorly understood, the fat-specific protein FSP27 has been shown to play a key role [9]. For a long time, LDs have been considered as passive cytoplasmic inclusions and only recently they have been recognized as dynamic organelles involved not only in lipid homeostasis and metabolism (as discussed above), but also in membrane trafficking, cell signalling, proliferation and apoptosis [8]. In this context, 3T3-L1 cells have been reported as a well-established model to study adipogenesis as they are converted into adipose-like cells under defined hormonal stimulation [10]. Adipocyte differentiation is characterized by sequential changes in the expression of specific genes, which determines the defined adipocyte cell phenotype [11]. During growth, preadipose cells resemble fibroblasts; upon reaching confluence, proliferative preadipocytes become growth-arrested by contact inhibition. After hormonal induction, these cells re-enter the cell cycle, then they stop proliferating again, and finally undergo terminal adipocyte differentiation, with morphological conversion into cells with a spherical shape and increased LDs accumulation [2,12]. The mechanism underlying the accumulation of neutral lipids into LDs is not yet well-defined and represents one of the major challenges in understanding the roles of lipids in biological and pathological processes.

The current imaging techniques applied to study cellular lipid dynamic rely on fluorescence microscopy upon staining with neutral-lipid dyes [1,13], which are, however, only applicable to fixed samples and subject to variability depending on the experimental conditions [14]. Unfortunately, these fluorescent dyes are often nonspecific and interfere with the lipid-mediated biological processes introducing imaging artifacts in cell recordings (e.g., may cause changes in LDs fusion behaviour) [13,14]. Moreover, fixation methods can dramatically impact on LDs morphology and on the detection of some LD-associated proteins after cell permeabilization. For long-term imaging, photobleaching events reduce the signal-to-noise ratio and consequently the quality of the images and quantitative data analysis. Indeed, more appropriate controls are required to discriminate false positive fluorescence signals. Thus, optical label-free imaging techniques need to be developed to overcome the limitation of the current methods for imaging of lipids, lipid-protein complexes and lipids cellular mobilization [15].

In this framework, vibrational microscopy has emerged in the last decade as a powerful alternative approach based on chemically label-free selective contrast generated by the intrinsic molecule vibrations. Among the vibrational techniques, spontaneous Raman is a widely-used label-free approach that detects specific chemical bond vibrations of molecules by inelastic light scattering. Thus, a typical Raman spectrum gives information on the molecular contents and chemical structure of the sample, offering an intrinsic chemical selectivity [16,17]. Unfortunately, spontaneous Raman microscopy is limited by weak signals requiring very long acquisition times, that limit severely its application to the study of living systems. To overcome this limit, label-free microscopy techniques based on nonlinear optics are rapidly gaining interest and are widely applied [18,19].

Stimulated Raman scattering (SRS) was one of the first nonlinear optical phenomena to be discovered [20]; however, it was adapted to microscopy only in the last decade [21–26]. Some years ago, it has been demonstrated that SRS signal can be further improved by one order of magnitude when femtoseconds (fs) pulses are used instead of picoseconds (ps) [27]. Finally, as a consequence of nonlinear excitation, SRS offers intrinsic 3D resolving capabilities of lipids within biological samples [28–30]. Thus, the use of SRS microscopy could overcome the current limitations and artefacts associated with fluorescence lipid staining [31].

SRS has proven to be particularly powerful for studying lipid-rich structures in diverse samples such as artificial model systems, living cells and tissues [23]. Up to now, a lot of SRS microscopy implementations are based on the interaction of a pair of Fourier transform-limited (FTL) picosecond (ps) laser sources. This approach enhances the contrast of SRS signals ensuring a high spectral resolution (*∼*10 cm^*-*1^) very useful in the fingerprint region (~800 and 1800 cm^-1^), where Raman peaks are narrow, closely spaced, and may be in abundance for a particular chemical [32].

In order to enhance spectral resolution in SRS microscopy based on FTL fs laser pulses, a number of methods have been developed, which relies on optical phase control. A feasible option is to impose a quadratic spectral phase variation; in other words, a linear variation of the frequencies within the pulse, called ‘chirp’. Laser pulses can be stretched temporally by a stretching factor *F* to τ = *F*τ_0_, where τ_0_, is the FTL pulse duration, while the instantaneous spectral bandwidth results be narrower than the FTL spectral bandwidth by a factor of 1/*F* [32]. By equally chirping pump and Stokes beams, it is possible to adjust the instantaneous frequency difference and its bandwidth to certain Raman linewidths and, in the limiting case, even mimic the picoseconds SRS scheme. This approach is referred to as spectral focusing (SF) [33–36]. However, the vast number of parameters that need to be considered when selecting and aligning the optics required for chirp-matching can be discouraging. Furthermore, due to fluctuations in the pump and Stokes wavelengths and dispersion in the microscope, perfect chirp-matching can be difficult to maintain. Thus, SF-SRS setups often have poorer spectral resolution than theoretically predicted [37,38].

SRS microscopy with ps laser pulses is equally successful at imaging in carbon-hydrogen (CH) stretching region (2800–3100 cm^−1^) where the molecular specificity is assumed to be low because lipids and proteins have a large Raman spectral shape (about 100 cm^-1^) and the difference between the CH_2_ and CH_3_ peaks is 95 cm^−1^. In addition, we note that while ps coherent Raman scattering guarantees the best spectral resolution, the optimal ratio of image contrast and signal intensity is reached when the spectral resolution matches the width of the Raman lines under consideration (5–100 cm^-1^) [39]. Because excitation with picosecond pulses can only match the linewidths in the fingerprint region (5–20 cm^-1^), the question is raised, as to whether broader bandwidth femtosecond (fs) pulses might also be well suited to optimal excitation of CH stretching vibrations (lipid and proteins linewidth ∼100 cm^-1^) [40].

In this study, we applied SRS microscopy to image the distributions of LDs in 3T3-L1 cells at different stages of adipocyte differentiation, in absence of exogenous labels. An increase in the number of large (>15μm^2^) lipid droplets and, assuming an elliptical arrangement of LDs around the cell nucleus, an increase of their major and minor axes lengths are demonstrated in human adipocyte cells during differentiation process. In addition, exploiting the laser pulses chirping due to their propagation and interaction with the optical elements of our set up (see SRS imaging paragraphs), the simultaneous visualization and spatial mapping of protein and lipid contents in a multicomponent system are confirmed by the separation of the measured CH_2_ and CH_3_ stretching signals. We note that being the chirping not controlled, the spectral resolution is not optimised, but as significant advantage we have that no further optical elements have to be introduced in our experimental set up. Finally, the three-dimensional sectioning capability of the SRS technique, performed by collecting SRS images at different focal plane along the z axis, is proved by a three-dimensional (3D) reconstruction of lipids, proteins and their simultaneous spatial distributions inside cells. Our findings confirm that stimulated Raman imaging provides an advanced label-free approach to image and follow changes in LDs potentially under pathophysiological conditions. This SRS application will provide a useful tool for diagnosis, analysis and follow-up after treatment for lipid-associated pathologies.

## Methods

### Cell culture

3T3-L1 cells (American Type Cell Culture, ATCC) were grown in Dulbecco's Modified Eagle's Medium (DMEM, ATCC) supplemented with 10% fetal bovine serum (Gibco), 100 U/mL penicillin and 100 mg/mL streptomycin in 8% CO_2_/ humidified atmosphere at 37°C. Differentiation to adipocytes was induced 2 days post 80% confluence by incubating the cells in the growth medium supplemented with 0.5 mM isobutylmethylxanthine (IBMX, Sigma), 1 μM Dexamethasone (Sigma) and 1 μg/mL insulin (Sigma) for 60 h, and then maintained in the same medium without IBMX and dexamethasone [8,10]. This medium was replaced every 2 days. Cells were used at different time points (0, 5, 10, 15 days) during the differentiation process from preadipocytes to mature adipocytes. At each time point the cells were fixed with 4% (w/v) paraformaldehyde in Phosphate-buffered saline (PBS) (supplemented with 50 mM NH_4_Cl to quench possible free aldehydes autofluorescence) for 10 min at room temperature and then processed for immunofluorescence procedures or for SRS imaging.

### Immunofluorescence procedures

Lipid droplets were stained with 10 μg/mL of BODIPY 493/503 or with HCS LipidTox Deep Red neutral lipid stain (1:125 dilution) or with Oil Red-O (1% Oil Red-O stock solution diluted 3:2 with water) for 30 min at room temperature. Following neutral lipid staining, cells were extensively washed with PBS and the stained lipid droplets were visualized and acquired using a Zeiss LSM700 laser-scanning confocal microscope with a 63× oil-immersion objective (Zeiss, Jena, Germany). Nuclei were stained with Hoechst 33258.

### Cellular protein extraction and Western blot analysis

Western blot analysis was performed to evaluate PPARγ and FABP4 protein expression levels in undifferentiated and differentiated 3T3-L1 cells. Briefly, 3T3-L1 preadipocytes were seeded onto six-well plates and induced to differentiate into adipocytes at the indicate time points. The cells were then collected and resuspended in lysis buffer [25 mM Tris, pH 7.4, 150 mM NaCl, 5 mM EDTA, 5 mM MgCl_2_, 10 mM NaF, 40 mM β-glycerophosphate, 1 mM Na3VO4, 1 mM dithiothreitol] supplemented with 1% (w/v) Triton X-100 and protease inhibitor mixture (30 min, 4°C, shaking). The lysates were centrifuged (13,000× g, 10 min, 4°C), with the supernatants assayed for protein concentration (Bradford assay) and immediately used. Thirty micrograms of proteins were separated by SDS–PAGE and transferred onto nitrocellulose membranes (Millipore). The membrane was blocked with 5% (w/v) skim milk powder (Sigma-Aldrich) in TBS-T [0.05% (w/v) Tween 20, 150 mM NaCl, 20 mM Tris-HCl, pH 7.5] followed by incubation with primary antibodies against PPARγ (Santa Cruz Biotechnology: sc7196, 1:500) and FABP4 (Santa Cruz Biotechnology: sc271529, 1:1,000) and then with secondary antibody (HRP-conjugated anti-rabbit or anti-mouse IgG, respectively). β-actin is used as internal protein levels.

### SRS microscope

Our SRS spectroscopy set-up is similar to the figure shown in [41] (see also S1 Fig). This system is a combination of a femtosecond SRS spectroscopy set-up with an inverted optical microscope (Eclipse TE-2000-E, Nikon) equipped with mirrors scanning unit (C2, Nikon). The two pulsed laser source are: (i) a femtosecond Ti:Sa (Chameleon Ultra II) with ≈140 fs pulse duration according to manufacturer datasheet, 80 MHz of repetition rate and 680–1,080 nm emission wavelengths range; (ii) a femtosecond synchronized optical parametric oscillator (SOPO-Chameleon Compact OPO), pumped by a Ti:Sa, with ≈ 200 fs pulse duration according to manufacturer datasheet, 80 MHz of repetition rate and 1,000–1,600 nm emission wavelengths range. This combination of laser systems allows a minimum photon energy difference between Ti:Sa and SOPO beam of 2,500cm^-1^; hence, only the high frequency C–H region (2,800–3,200cm^-1^) of Raman spectra can be explored. Immediately following emission from the laser, the intensity of the Ti:Sa pulses are modulated at a frequency of 4.5 MHz (thus, faster than the typical laser noise, e.g. 1 MHz) by an EOM (CONOPTICS 350–160 KD*P) pulse selection system, allowing to have high sensitivity SRS signal detection at the moderate laser power required for our biological imaging. Additionally, due to a slightly different path of the two laser beams inside the OPO housing, they have a time delay of about 5 ns. In order to generate a high sensitivity SRS signal, the pump (Ti:Sa) and probe (SOPO) pulsed lasers have been spatially and temporally overlapped at the image plane. Therefore in the system were inserted: (i) a delay line (Newport MOD MILS200CC) between the Ti:Sa and the microscope in order to obtain a temporal overlap; and (ii) a dichroic mirror (Semrock FF875-Di01-2536) to spatially combine the collinear beams. The two beams were then focused into the specimen through a 60× multiphoton microscope objective (NA = 1.27). The output pulses are collected in transmission by a 40× high numerical aperture multiphoton microscope objective (NA = 1.25). In order to remove the pump signal, a stack of optical filters was used, while the probe signal is measured by a photodetector (PD). The PD output is connected by a 50 Ω low pass filter to a lock-in amplifier (LIA, SR844-200MHz dual phase), thus the readout of the PD is demodulated by the LIA to extract the modulation depth. The focused power was less than 10 mW for both pump and probe beams. The lock-in amplifier (LIA) time constant was set to 100 μs with a slope of 18dB/oct and a sensitivity of 10 μV.

In our microscope system, two-dimensional (2D) imaging is realized through beam scanning with 2D galvo mirrors by a sequential collection of pixels, which are acquired and quantized in intensity by a data acquisition chain. A 2D image is obtained through the synchronization of the whole system (i.e. the forward detection unit with the microscope scanning unit). The synchronization is achieved by managing: (i) the PCI card (NI PCIe 6363) through an in-house LabVIEW program; (ii) the electrical signal detected by LIA; and, (iii) the digital signals provided by the microscope scanning unit controller. All images were analysed with ImageJ software (Rasband, W.S., National Institutes of Health, Bethesda, Maryland, USA) and three dimensional images were realized by the plugin “3D Volume Viewer”.

## Results and discussion

### Fluorescence imaging

**Fig 1:**
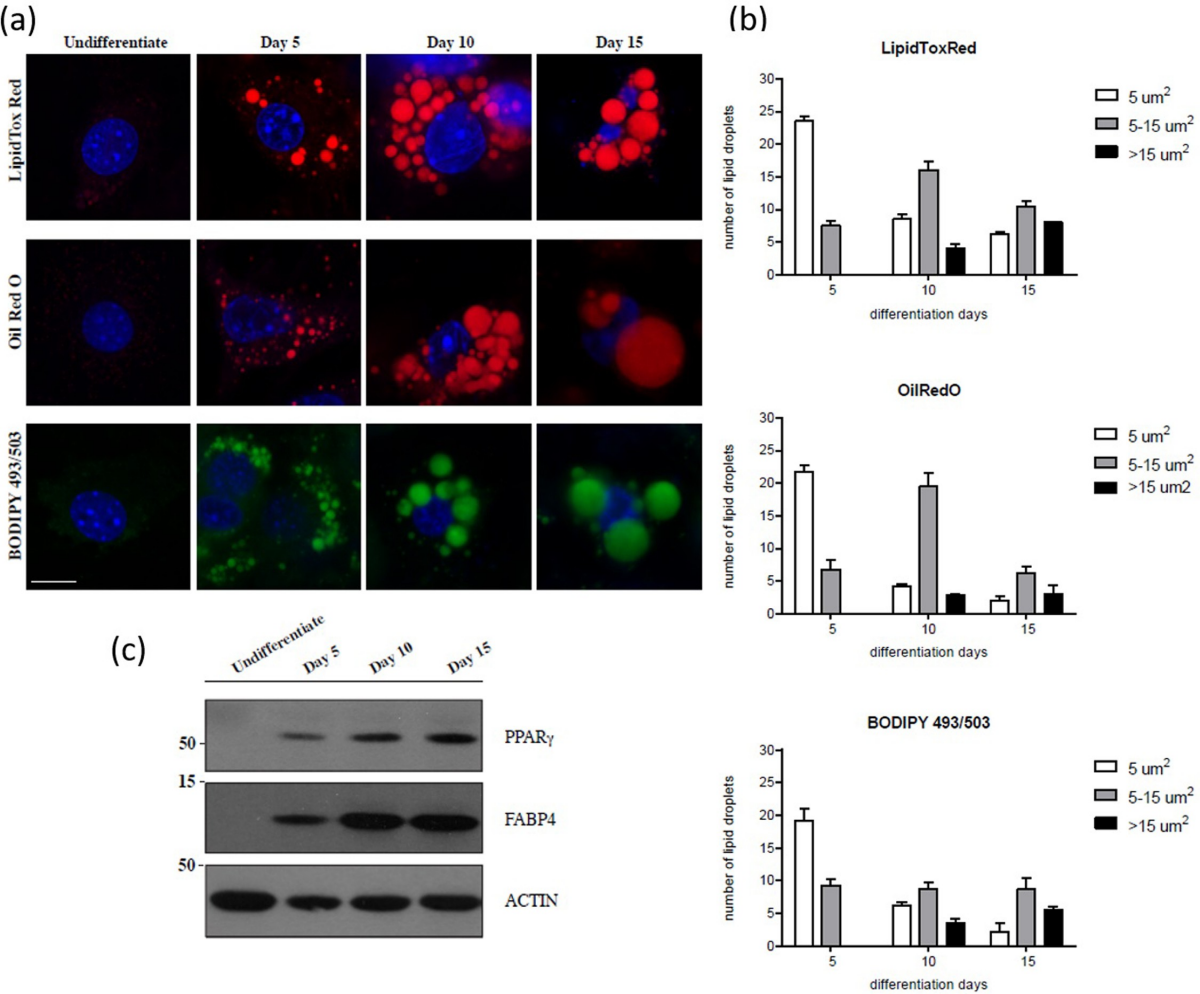
Fluorescence imaging. a) Representative confocal microscopy images of 3T3-L1 cells Undifferentiated or differentiated with cultured adipogenic media, fixed at day 5, day 10 and day 15 of the differentiation process, fixed and labeled with LipidTox Red (red), Oil Red O (red) or BODIPY 493/503 (green) fluorescent dyes. Cell nuclei were labelled with DAPI (blue). Scale bar, 10 μm. b) Quantification of LDs size (as area ranges, μm^2^) in 3T3-L1 cells treated, fixed and labeled as in (a) (Experimental data are reported in S1 File). c) Western blotting with anti-PPARγ and anti-FABP4 antibodies (as indicated) of total cell lysate (20 μg/lane) from 3T3-L1 cells treated as in (a). Actin is shown for the internal protein levels. Molecular weight standards (kDa) are indicated on the right of each panel. Of note, PPARγ is separated on 10% SDS-PAGE while FABP4 is separated on 15% SDS-PAGE (S2 Fig).

Adipocytes are readily identified by the accumulation of LDs through the differentiation processes. The differentiation of the 3T3-L1 cellular system is commonly used in studies on adipogenesis and on LDs biogenesis. The most frequently used pro-differentiative agents are insulin, dexamethasone, and 3-isobutyl-1-methylxanthine (IBMX) at concentrations of 10 μg/mL, 1 μM, and 0.5 mM, respectively [2,12,13]. Approximately 5 days after addition of these agents to 3T3-L1 cells in culture, these started accumulating lipids into LDs that grew in size over cultivation time (Fig 1A). We first visualised this differentiation process by immunofluorescence under confocal microscopy, using fluorescent lipophilic dyes, which partition into the nonpolar LDs core and are widely used as markers for the detection of LDs [14]. Here, we tested the most commonly used lipophilic dyes LipidTox Red, Oil Red O and BODIPY 493/503 to image the LDs at 5, 10 and 15 days of the differentiation process and, for comparison, we observed also the undifferentiating control cells (Fig 1A and 1B). Intracellular LDs started to appear after 5 days and over the following days they increased in size and decreased in number (Fig 1A). Unfortunately, the visualization of LDs by immunofluorescence has several methodological limitations [42]. Even though the three fluorescent dyes employed have been widely developed to visualize the intracellular lipid stores by confocal microscopy, each of them has its own strengths and weaknesses. For instance, LipidTox Red gives a good fluorescent signal of LD membranes and, although it bleaches faster, has a better staining efficiency than Oil Red O. By contrast, the green fluorescence of BODIPY 493/503 is moderately specific for LDs and the fluorescent signal generally appears less intense than that of Oil Red O [14,15] (Fig 1A).

In order to correlate the adipogenic differentiation to the immunofluorescence imaging, we examined the protein expression profile of two master regulators of the differentiation processes and metabolism: peroxisome proliferator-activated receptor γ (PPARγ) and fatty acid binding protein 4 (FABP4) (Fig 1C). Both PPARγ and FABP4 expression levels increased from day 5 to day 15 (Fig 1C), and this correlated with the temporal formation and maturation of LDs in cells with culture adipogenic media, as detected by confocal microscopy (Fig 1A). Of note, the expression levels of PPARγ and FABP4 were almost undetectable in undifferentiated conditions (Fig 1C).

It should be pointed out that the use of lipophilic dyes, although informative, has some limitations: they are unable to discriminate the chemical composition of the sample and the fluorescence intensity readouts are non-quantitative due to the contribution of photobleaching.

### SRS imaging

To overcome the above mentioned limitations of fluorescence imaging, we applied a non-invasive, non-destructive and label-free microscopy platform which could determine intermediate cell states by utilising chemically-specific vibration imaging based on SRS. In SRS, the sample is excited by two collinear laser beams (pump and Raman signal) at different frequencies. If the difference in frequencies is identical to a molecular vibration of the sample, a coherent excitation of molecular bond vibration modes occurs and a significant increase of the Raman signal is obtained. In this case, Raman signal, differently from linear Raman scattering, is a coherent radiation characterized by nonlinear dependence on the incoming light fields, that allows vibrational contrast mechanism for fast imaging with high spectral and spatial resolution and improved signal-to-noise ratio [43]; this offers an intrinsic three-dimensional (3D) spatial species resolution and sectioning [28–30,44].

In our experiments, the stimulated Raman gain (SRG) modality was carried out, i.e. the intensity gain of the Stokes beam due the excitation of molecular vibrations in the focus was measured (see methods section). All the images, acquired under fixed experimental conditions, were single recordings of 512px × 512px with an acquisition time of 16s.

**Fig 2:**
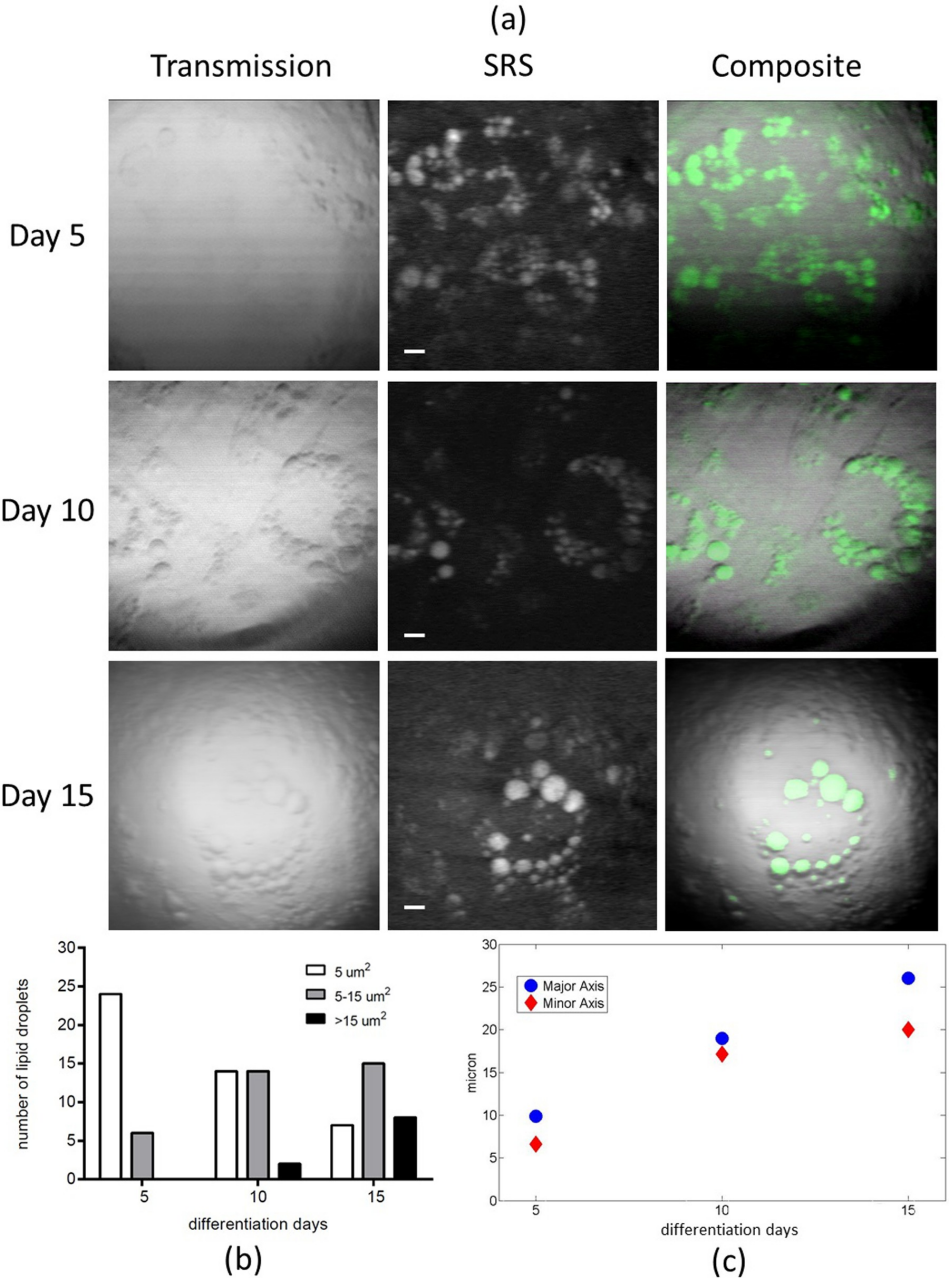
Subcellular localization of lipid droplets in adipocyte cells by SRS imaging. a) Representative transmission microscopy images (first column) and SRS microscopy images acquired at 2,845 cm^-1^ (second column) of adipocyte cells, fixed at day 5, day 10 and day 15 of the differentiation process. Merged images are reported in the last. All scale bars are 5 μm. b) Quantification of LDs size (as area ranges, μm^2^) in adipocytes at day 5, day 10 and day 15 of the differentiation process (as indicated); experimental data are reported in S2 File. c) Quantification of major and minor axis (μm) of the fitted ellipsis in which LDs are arranged in adypocytes fixed at day 5, day 10 and day 15 of the differentiation process (as indicated); experimental data are reported in S3 File.

In order to image LDs and to investigate their maturation at different times, the 2,845 cm^-1^ CH_2_ stretching mode was investigated. The pump beam was set at 810 nm and the probe wavelength was set at 1,053 nm. SRS images, acquired at a fixed focal plane by a 2.5x scanning zoom, of day 5, day 10 and day 15 samples, are shown in the middle column of Fig 2A. Each SRS image was obtained by averaging ten images acquired under the same experimental conditions. LDs and their distribution inside adipocytes were clearly visualized (Fig 2A). To better point out the effect of differentiation process, in the first column of Fig 2A the transmission images of day 5, day 10 and day 15 samples are reported, while in the last column of Fig 2A the merge image of two modalities, i.e. transmission and SRS images, are shown, too.

As shown in Fig 2B, a high number of small LDs (<5μm^2^) and only few medium (5–15μm^2^) LDs were detected in the day 5 sample, while in day 10 and day 15 samples, a decrease in the number of small (<5μm^2^) LDs and a parallel increase in the number of medium (5–15μm^2^) and large (>15μm^2^) LDs were measured. Results obtained are in agreement with the growing trend found by standard fluorescence analysis reported in Fig 1B. Moreover, since results obtained with BODIPY 493/503 fluorescent dye are those that best match the corresponding statistical analysis performed on SRS images in a label free approach, we can conclude that BODIPY 493/503 seems to have a lower influence on the maturation of LDs. This is not a surprise, indeed several reports showed and discussed the artifactual effects on LD morphology upon fixation and staining with Oil Red O dye [45,46]. In addition, assuming an elliptical arrangement of all the LDs around the cell nucleus, major and minor axes were identified and a linear increase in their lengths was measured from day 5 to day 15 (Fig 2C).

Currently, our implemented SRS microscope allows to probe a single Raman band at a time. Different Raman bands, corresponding to different chemical components of the sample, can be imaged by tuning the frequency either of the pump or the Stokes beams in sequential scans, allowing the detection of one chemical contrast for each scanning. Thus, CH_2_ (2,845 cm^-1^) and CH_3_ (2,940 cm^-1^) stretching signals can be collected at one Raman shift at a time, leading in principle to map the distributions of the lipid and protein contents on the same field of the sample. We note that in our experimental set up, the full width at half maximum (FWHM) of Ti:Sa and OPO cross correlation, measured by an auto/cross correlator (pulseCheck A.P.E.) before the microscope, was of 253 fs (see S3 Fig and S4 File). This corresponds to an experimental spectral bandwidth, given by the FWHM of cross correlation in the frequency domain [36], of 61 cm^-1^. As a consequence, when we tune the lasers beams to excite the 2,845 cm^-1^ band, actually we excite the range (FWHM) 2,818–2,879 cm^-1^; similarly, when we tune the lasers beams to 2,940 cm^-1^, we are exciting from 2,908 to 2,970 cm^-1^ (FWHM) (see S4 Fig). In addition, due to the propagation inside the scan head and microscope objective, a significant further chirping of laser pulses and, as a consequence, an improvement of spectral resolution are both expected at the sample. Definitely being our experimental spectral resolution (about 60 cm^-1^) less than the linewidth of the Raman band of lipids and proteins (100 cm^-1^), we can conclude that our experimental set up can be suitable for imaging applications that probe for molecular specificity such as lipids, proteins in C-H region.

Moreover, when fs pulses are used, since the loss in spectral selectivity of SRS signal is not prejudicial in lipids imaging in the broad spectral range of CH bond vibrations [27], SRS images acquired at 2,845 cm^-1^ can be mainly attributed to the lipids. On the contrary, the chemical specificity of SRS images at 2,940 cm^-1^ is not particularly efficient. Indeed, since both lipids and proteins have significant Raman signals and their Raman bands are partially overlapped, SRS images acquired at 2,940 cm^-1^ contain both lipids and proteins signals. To distinguish lipids and proteins content, since for these biological samples the calibration of individual components is not available, we subtract the CH_2_ from the CH_3_ image such that the CH_2_ signal vanishes and the resulting difference image shows only proteins signal [40,47–49] (the analytical study for the separation of lipids and proteins content is showed in S5 Fig).

**Fig 3:**
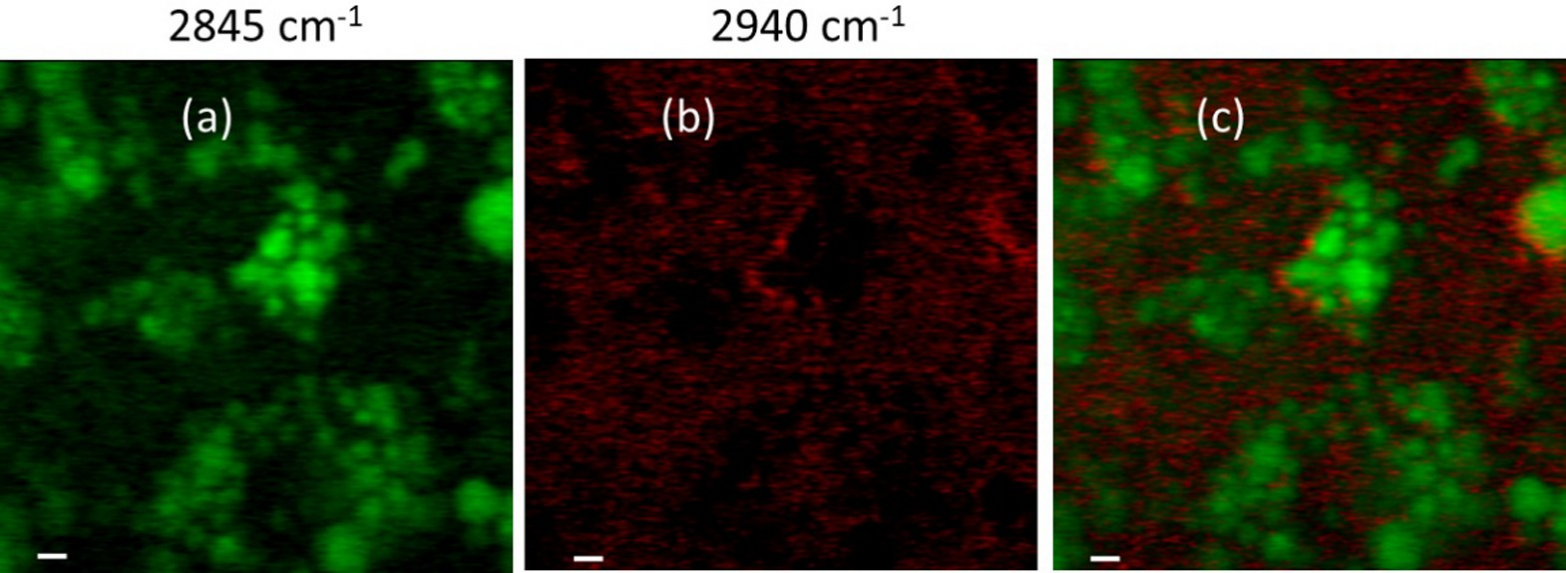
Representative single focal plane of Z stack SRS image of adipocytes fixed at day 5 of the differentiation process. Images acquired with a 2.5x optical zoom at: a) 2,845 cm^-1^ (lipids contribution); b) 2,940 cm^-1^ (proteins contribution, retrieved image). c) Lipids (green) and proteins (red) contributions in cells acquired as in a-b. Scale bars, 5 μm.

In Fig 3A, the SRS image of day 5 sample, acquired at 2,845 cm^-1^ and at a fixed focal plane with a 2.5x scanning zoom is reported. Concerning the SRS image at 2,940 cm^-1^, the pump beam was set at 810 nm and the probe beam wavelength was tuned at 1,063 nm. In Fig 3B, the successfully retrieved protein distribution map in adipocytes is reported (the acquired and unprocessed image is reported in S5B Fig). The simultaneous distribution of proteins (red) and lipids (green) inside adipocytes, obtained by merging Fig 3A and 3B, is shown in Fig 3C.

**Fig 4:**
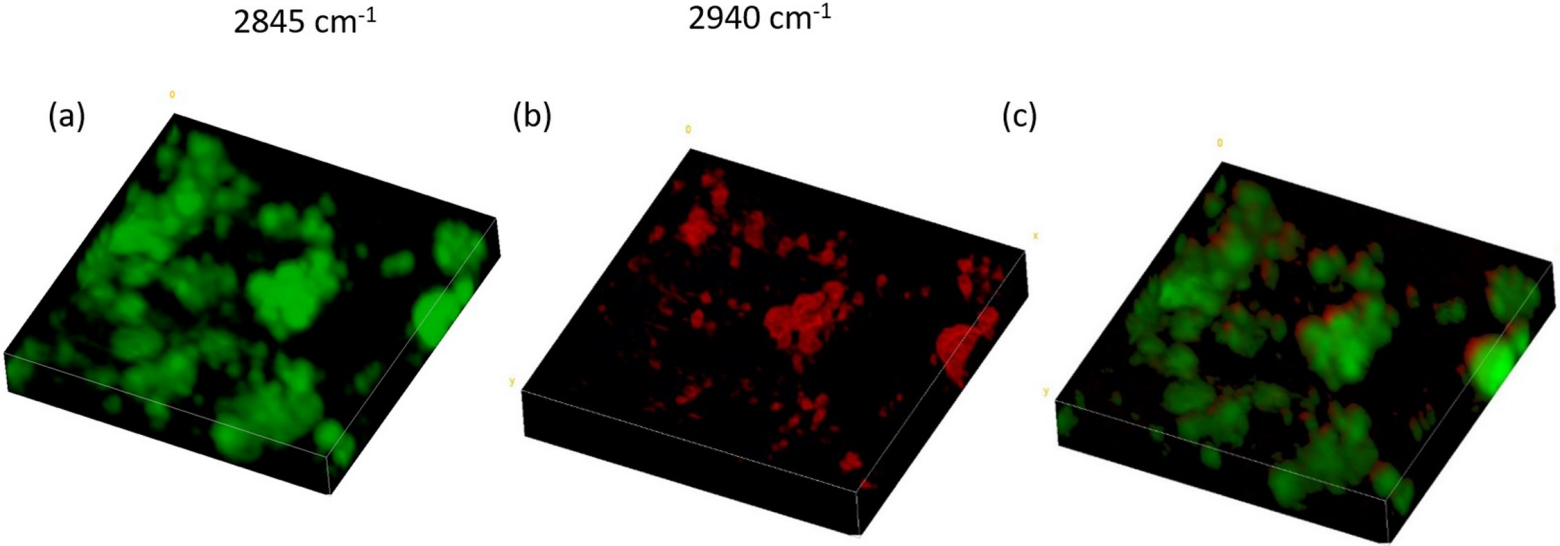
Three-dimensional reconstruction of lipids, proteins and their simultaneous distribution in adipocytes fixed at days 5 of the differentiation process. a) three-dimensional Z stack (13 frames, 1 μm/step) reconstruction of SRS images at 2,845 cm-1; b) three-dimensional reconstruction of SRS images at 2,940 cm-1; c) three-dimensional reconstruction of simultaneous lipids and proteins distribution. All the images are acquired with a 2.5x optical zoom.

A 3D volume reconstruction for day 5 sample for both lipids and proteins was performed, acquiring a z stack (number of frames = 13, step = 1 μm) of SRS images at 2,845 cm^-1^ and at 2,940 cm^-1^, respectively (Fig 4A and 4B). The 3D composite image in Fig 4C was achieved by merging the two previous SRS images, where proteins are displayed in red and lipids in green.

**Fig 5:**
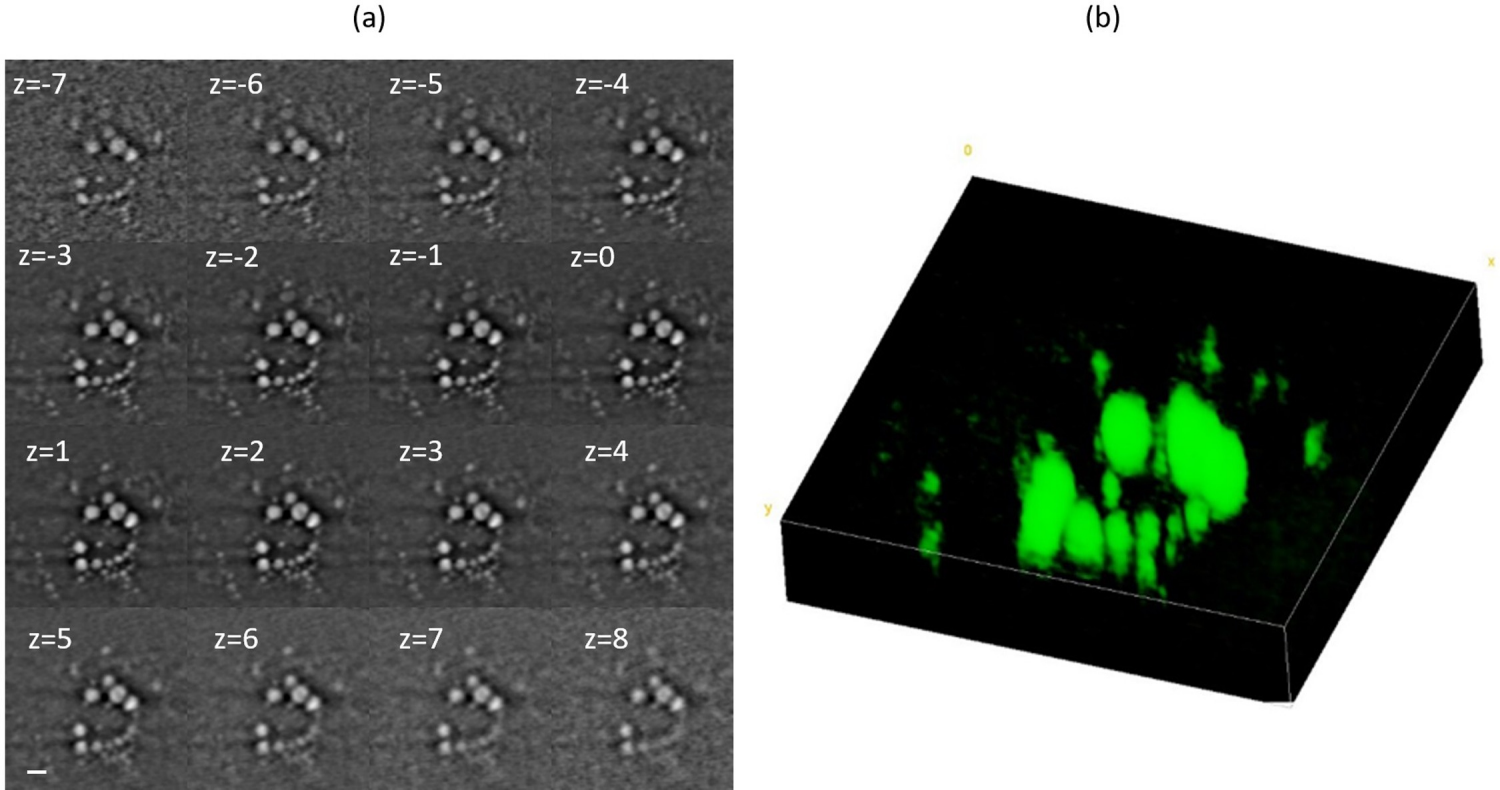
SRS z-stack for 3D volume reconstruction of lipids. a) Single representative z-sections (16 frames, 1 μm /step) of SRS images at 2,845 cm^-1^ of adipocyte fixed at day 15 of the differentiation process. b) three-dimensional reconstruction of lipids.

Finally, Fig 5A shows SRS images at 2,845 cm^-1^ for day 15 sample acquired at different focal planes achieved by a fine z-movement (number of frames = 16,) steps of 1 μm. These frames have been used for 3D volume reconstruction of lipids (see Fig 5B).

## Discussion

The correct homeostasis of lipid metabolism is essential for life and dysregulation of this metabolism is associated with severe human diseases including cancer [3]. The increased prevalence of lipid-related pathologies in the last several years, has promoted the study of the mechanism involved in adipocyte differentiation and adipogenesis. These are complex processes that can be analysed thanks to the recent development of different cell models and biochemical and biophysical techniques [50].

One of the most common cellular model system to study adipogenesis is the 3T3-L1 cell line, which can be converted from its fibroblast phenotype into adipocytes following treatment with adipogenic agent. In the last five years this cellular system has been used to evaluate the effects of compounds or nutrients on adipogenesis and in the treatment of obesity, as well as to study the function of different genes of the inflammatory pathways, adipokine synthesis, and cellular secretion in adipogenesis [50,51]. All these studies have so far relied on invasive methods; therefore, a reliable non-invasive, label-free and non-destructive imaging technique could be of great value to characterise adipocyte morphology and function.

With this aim, in this study SRS has been successfully used for lipids investigation, giving several advantages such as: low photodamage, low phototoxicity and no photobleaching [52–54]. Indeed, a lipids milieu is characterized by large amounts of fatty acid chains with CH bonds and specific CH_2_ groups whose relative peaks in their Raman spectrum are associated with CH bond vibrational states at 2,845 cm^-1^; this provides a unique fingerprint signature for lipids inside the cell. Additionally, LDs provide a very strong SRS signal suggesting that this technique can be used to investigate many unresolved questions on lipid-related processes in cells and tissues [52–57]. Moreover, in several biological and biomedical applications, it is desirable to map simultaneously the different chemical species that compose a complex system such as a tissue. SRS is a powerful method to map in a label-free manner the distributions of individual species in a multicomponent system. This is due to the linear dependence on the individual specie concentrations of SRS signals and to the chemical selectivity provided by this technique [48,58–60].

SRS imaging allows to achieve higher definition on lipid accumulation within adipocytes compared to traditional fluorescent dyes staining approaches, namely LipidTox Red, Oil Red O and BODIPY 493/503. We report the capability of SRS imaging to evaluate and determine, during the differentiation process, the changes in number and dimension of LDs and their intracellular redistribution from the nucleus to the cell periphery following a roughly elliptical crown. These results assess the ability of this nonlinear imaging technique to monitor the maturation of LDs during the adipocyte differentiation. Then, by further implementing a temporal multiplexing to sequentially acquire the two channels, corresponding to CH_2_ (lipid; 2,845 cm^-1^) and CH_3_ (protein; 2,940 cm^-1^) stretching vibrational signals, we were able to collect the chemically specific spectral differentiation of SRS imaging, even if the two bands partially overlap. We should emphasize that SRS imaging of proteins in adipocytes is challenging, because of the lower protein density with respect to the lipids [54,61].

Due to its nonlinear nature that confines the signal generation in the focal volume, SRS microscopy allows a three-dimensional sectioning similar to that of multiphoton fluorescence microscopy. In our study, we report the capability of SRS microscopy technique to develop the chemical decomposition of a single adipocyte cell into protein and lipid components, and to provide a two-colour three-dimensional imaging with high spatially resolved concentration maps of the two components.

## Conclusions

With the present study we have demonstrated the useful application of SRS imaging to analyze and characterize adipocyte differentiation. We report the capabilities of SRS imaging technique to evaluate and determine, during the differentiation process, the changes in number and dimension of LDs. We also successfully demonstrate an increase in the number of large (>15μm^2^) lipid droplets; an increment in their major and minor axes lengths, assuming an elliptical arrangement of LDs around the cell nucleus; their intracellular redistribution from the nucleus to the cell periphery following a roughly elliptical crown; spatially-resolved maps of lipids and proteins inside cells; three dimensional reconstructions of lipids at the initial and final steps of adipocyte differentiation are reported, too. This successful use of SRS imaging technique establishes its future application in the real-time dynamics study of LDs in the cellular environment. The SRS will permit the elucidation of fundamental LD-associated biological processes such as LD formation, maturation, as well as, misregulation of their functions in pathophysiological conditions (e.g, obesity, diabetes, atherosclerosis, fatty liver diseases). In addition, SRS microscopy technique overcame the limitations of the fluorescent probes (such as; cell fixation and permeabilization, limited photostability, limited range of emission colors and Stokes shifts, signal background, cell toxicity) and will be applied in in-vivo studies to precisely imaging the localization, distribution and biophysical properties of LDs. This, in turn, will permit a long-term monitoring of LD-related biological processes associated with a spatio-temporal resolution.

## Supporting information

S1 Fig Stimulated Raman microscope set-up schematic. Schematic layout of the f-SRS microscope system. OPO = Optical Parametric Oscillator; Ti:Sa = Ti:Sapphire laser; M1–M5 = Mirror; DM1, DM2, = Dichroic Mirror; EOM = Electro-Optic Modulator, FG = Function Generator; GM = Galvo Mirror; PD = Photodiode; DAQ = Data acquisition system; PC = Personal Computer.
(TIF)

S2 Fig Full scan images of all Western blotting data. (TIF)

S3 Fig Auto/cross correlator measure. Measured (blue dot) and fit (blue line) pulses duration of Ti:Sa and OPO cross correlation. Measure was performed by an autocorrelator (pulseCheck A.P.E.).
(TIF)

S4 Fig Experimental spectral bandwidth. SRS spectral bandwidths for CH_2_ (2,845 cm^-1^, blue lines) and CH_3_ (2,940 cm^-1^, red lines) stretching signals. Dotted lines are obtained considering an initially unchirped (transform-limited) Gaussian pulse with a pulses duration of 140 fs and 200 fs. After propagating through dispersive materials, pulses are chirped giving a broadenig of the spectral bandwidth. Continuous lines are obtained considering the Ti:Sa and OPO cross correlation reported in S3 Fig. at the input of the microscope (i.e. 253 fs), thus a higher spectral bandwidth can be achieved with chirped pulses [34]. Orange area highlight the overlap between two excited bandwidths; however in this region the intensities are well below the FWHM values, thus under threshold, so they do not contribute to the Raman signal.
(TIF)

S5 Fig Analytical study for the separation of lipids and proteins content. SRS unprocessed images of 3T3-L1 cell at day 5 of the differentiation process acquired at (a) 2,850, and (b) 2,940 cm^-1^. The intensity profiles along the dashed lines are shown for each SRS image. Intensity profiles across the same (c) horizontal and (d) vertical dashed lines in both 2,845 cm^-1^ acquired images reported in (a) and the same lines plotted in the retrieved proteins signal showed in Fig 3B and obtained by subtracting the CH_2_ from the CH_3_ image. Note the good complementarity in profiles of the two components in correspondence of LDs and their border and in the cytoplasm, thus protein and lipid are clearly distinguished with this linear combination calculation.
(TIF)

S1 File Experimental data for quantification of LDs size (as area ranges, μm^2^) in adipocytes at day 5, day 10 and day 15 of the differentiation process obtained by analyzing fluorescent images. (XLSX)

S2 File Experimental data for quantification of LDs size (as area ranges, μm^2^) in adipocytes at day 5, day 10 and day 15 of the differentiation process obtained by analyzing SRS images. (XLSX)

S3 File Experimental data for quantification of LDs elliptical arrangement in adipocytes at day 5, day 10 and day 15 of the differentiation process obtained by SRS imaging. (XLSX)

S4 File Experimental values of the measured cross correlation between Ti:Sa and OPO. (TXT)
