# Supplementary figures and images for: Three-dimensional label-free imaging throughout adipocyte differentiation by stimulated Raman microscopy
Source: PLoS One. 2019 May 21;14(5):e0216811. doi: 10.1371/journal.pone.0216811 (PMC6528968; doi:10.1371/journal.pone.0216811)

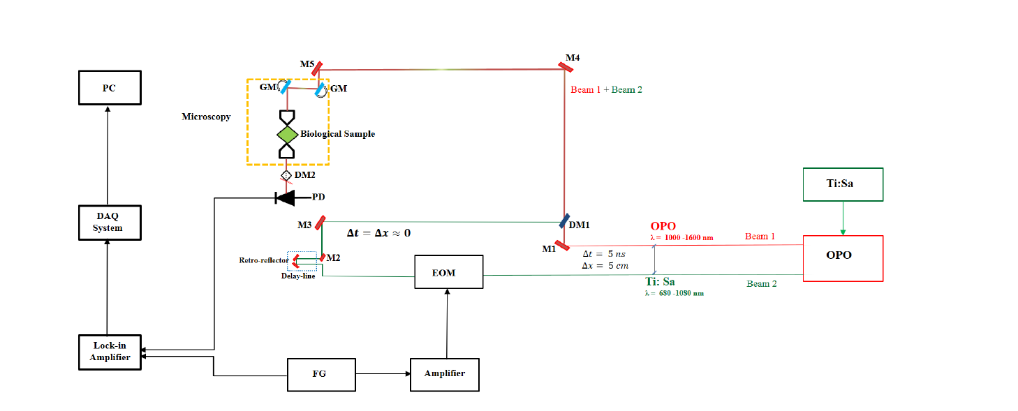

Supplement: S1 Fig — Schematic layout of the f-SRS microscope system. OPO = Optical Parametric Oscillator; Ti:Sa = Ti:Sapphire laser; M1–M5 = Mirror; DM1, DM2, = Dichroic Mirror; EOM = Electro-Optic Modulator, FG = Function Generator; GM = Galvo Mirror; PD = Photodiode; DAQ = Data acquisition system; PC = Personal Computer. (TIF) [file pone.0216811.s001.tif]

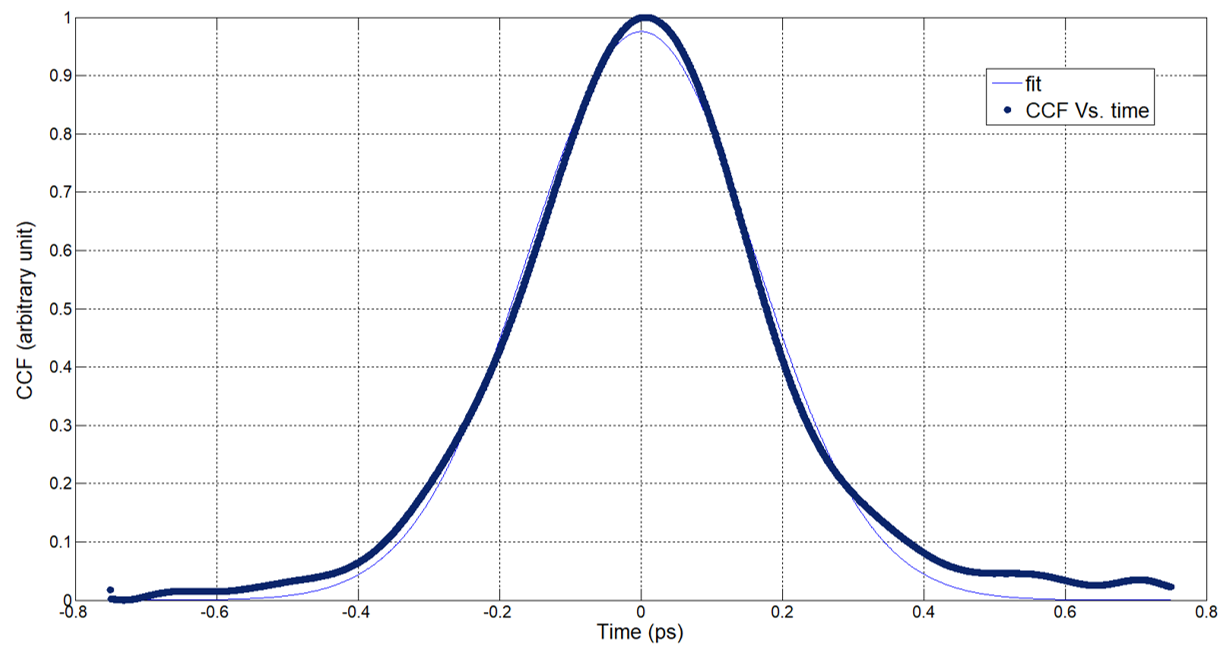

Supplement: S3 Fig — Measured (blue dot) and fit (blue line) pulses duration of Ti:Sa and OPO cross correlation. Measure was performed by an autocorrelator (pulseCheck A.P.E.). (TIF) [file pone.0216811.s003.tif]

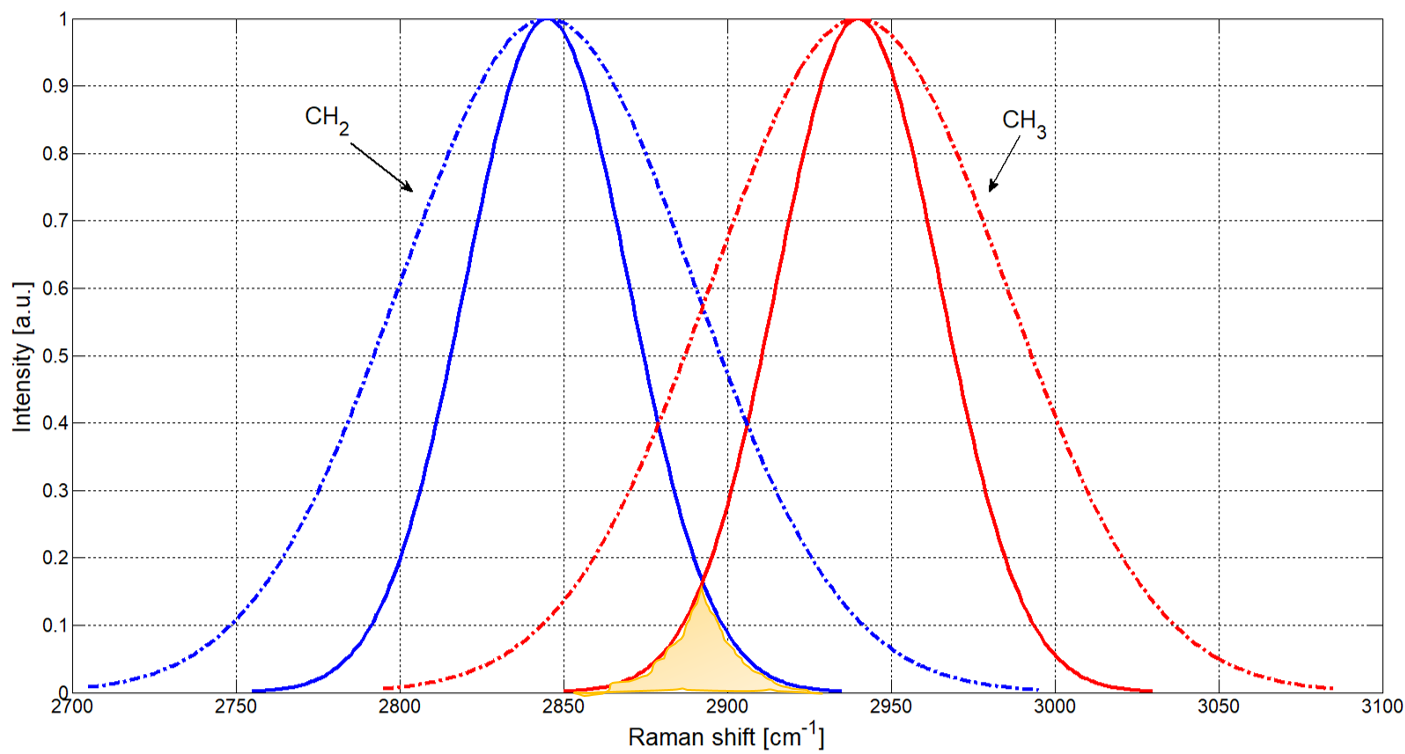

Supplement: S4 Fig — SRS spectral bandwidths for CH2 (2,845 cm-1, blue lines) and CH3 (2,940 cm-1, red lines) stretching signals. Dotted lines are obtained considering an initially unchirped (transform-limited) Gaussian pulse with a pulses duration of 140 fs and 200 fs. After propagating through dispersive materials, pulses are chirped giving a broadenig of the spectral bandwidth. Continuous lines are obtained considering the Ti:Sa and OPO cross correlation reported in S3 Fig. at the input of the microscope (i.e. 253 fs), thus a higher spectral bandwidth can be achieved with chirped pulses [34]. Orange area highlight the overlap between two excited bandwidths; however in this region the intensities are well below the FWHM values, thus under threshold, so they do not contribute to the Raman signal. (TIF) [file pone.0216811.s004.tif]

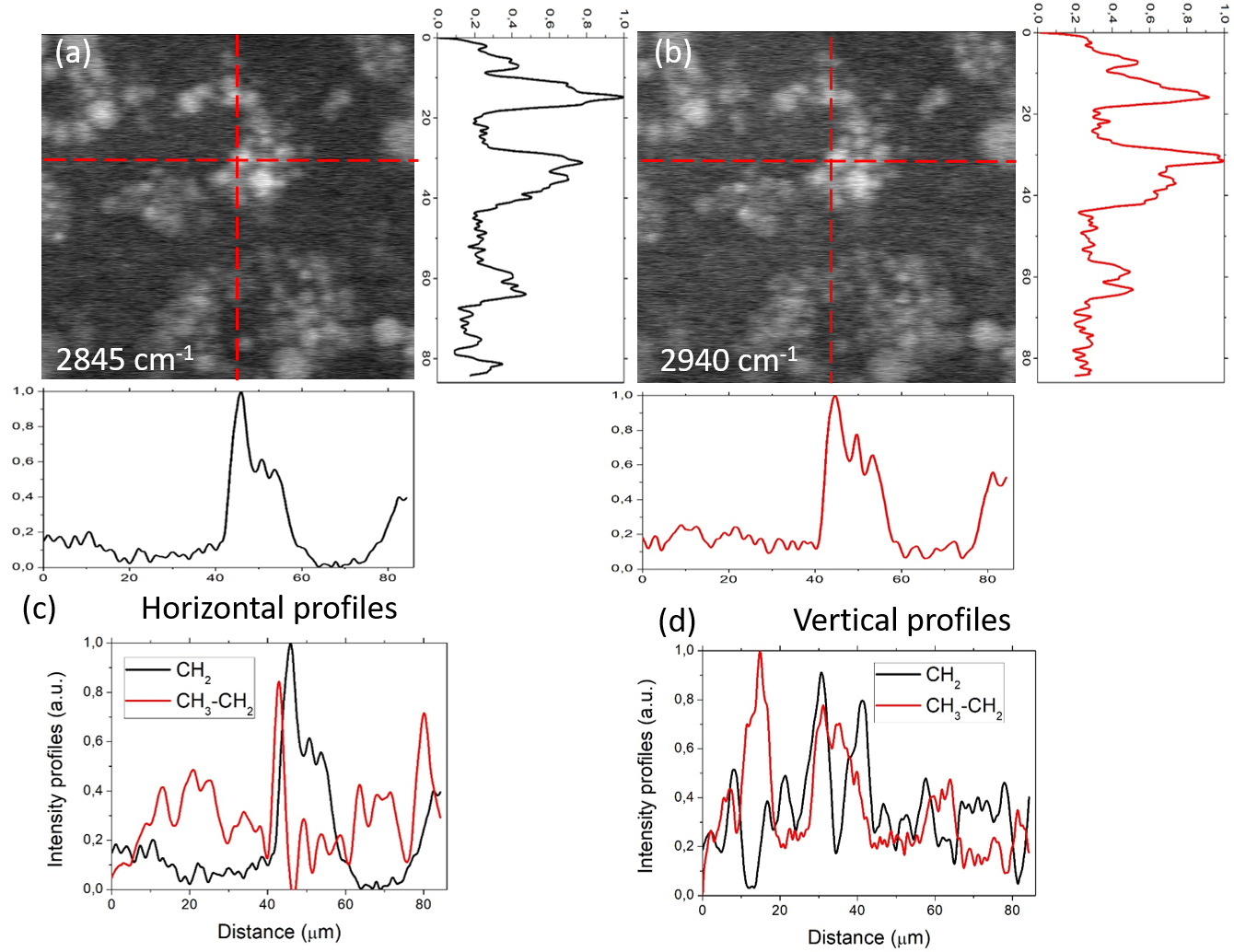

Supplement: S5 Fig — SRS unprocessed images of 3T3-L1 cell at day 5 of the differentiation process acquired at (a) 2,850, and (b) 2,940 cm-1. The intensity profiles along the dashed lines are shown for each SRS image. Intensity profiles across the same (c) horizontal and (d) vertical dashed lines in both 2,845 cm-1 acquired images reported in (a) and the same lines plotted in the retrieved proteins signal showed in Fig 3B and obtained by subtracting the CH2 from the CH3 image. Note the good complementarity in profiles of the two components in correspondence of LDs and their border and in the cytoplasm, thus protein and lipid are clearly distinguished with this linear combination calculation. (TIF) [file pone.0216811.s005.tif]
